# Deciphering microbial interactions and detecting keystone species with co-occurrence networks

**DOI:** 10.3389/fmicb.2014.00219

**Published:** 2014-05-20

**Authors:** David Berry, Stefanie Widder

**Affiliations:** ^1^Division of Microbial Ecology, Department of Microbiology and Ecosystem Science, University of ViennaVienna, Austria; ^2^CUBE-Division of Computational Systems Biology, Department of Microbiology and Ecosystem Science, University of ViennaVienna, Austria

**Keywords:** network analysis, microbial competition, microbial cooperation, 16S rRNA sequencing surveys, Lotka-Volterra models, keystone species, habitat filtering, correlation analysis

## Abstract

Co-occurrence networks produced from microbial survey sequencing data are frequently used to identify interactions between community members. While this approach has potential to reveal ecological processes, it has been insufficiently validated due to the technical limitations inherent in studying complex microbial ecosystems. Here, we simulate multi-species microbial communities with known interaction patterns using generalized Lotka-Volterra dynamics. We then construct co-occurrence networks and evaluate how well networks reveal the underlying interactions and how experimental and ecological parameters can affect network inference and interpretation. We find that co-occurrence networks can recapitulate interaction networks under certain conditions, but that they lose interpretability when the effects of habitat filtering become significant. We demonstrate that networks suffer from local hot spots of spurious correlation in the neighborhood of hub species that engage in many interactions. We also identify topological features associated with keystone species in co-occurrence networks. This study provides a substantiated framework to guide environmental microbiologists in the construction and interpretation of co-occurrence networks from microbial survey datasets.

## Introduction

The study of co-occurrence and co-abundance patterns has a long history in ecological research. In macro-ecological surveys non-random species co-occurrence patterns are often observed, indicating that community structure is imprinted by interactions between species. Fundamentally, interactions can be either positive or negative (Faust and Raes, 2012), and consequently lead to either aggregation or alternatively avoidance or exclusion (Cody and Diamond, 1975; Stone and Roberts, 1992; Gotelli and McCabe, 2002). Research on macro-ecological interaction networks and their topologies has revealed that community-wide interaction patterns maximize robustness and functionality (Montoya et al., 2006; Thébault and Fontaine, 2010; Saavedra et al., 2011). They are therefore fundamental units for understanding community dynamics and productivity.

Microorganisms also engage in a rich diversity of relationships. Interactions can be antagonistic, such as competition for a limiting resource or direct interference (e.g., by bacteriocin and siderophore production). Interactions can also be cooperative, such as transfer of complementary metabolites (e.g., interspecies H_2_ transfer) or quorum sensing (Hibbing et al., 2010). Because interactions can affect population dynamics it is expected that signatures of microbial interactions are imprinted in microbial survey datasets.

Knowledge about the composition of microbial communities from diverse environments is rapidly expanding due to tremendous advances in sequencing technologies. These technologies power ever broader and deeper surveys using targeted marker genes such as the 16S rRNA gene as well as shotgun metagenomics and metatranscriptomics (Caporaso et al., 2011; Shokralla et al., 2012). Even though the large data sets resulting from these surveys do not provide direct evidence on interaction between species, they are amenable to co-occurrence network construction using correlation coefficients or other association metrics (e.g., Ruan et al., 2006; Barberán et al., 2012; Eiler et al., 2012; Schwab et al., 2014). Ecological interpretations are widely applied to these co-occurrence networks, though validation of these interpretations remains scarce (Faust and Raes, 2012). This lack of validation is particularly problematic in microbial surveys because the physiological capabilities or ecological niches of the organisms under investigation can frequently not even be roughly predicted due to the lack of closely related cultivars or sequenced genomes (Stecher et al., 2013).

Most microbial communities are complex, consisting of many species potentially interacting with each other. This makes validation of community-wide interactions difficult if not impossible. In some cases experimental validation can be undertaken by constructing assemblages of organisms and inferring interactions based on combinations of these assemblages (Trosvik et al., 2010). This approach is limited, however, because constructed assemblies may not be relevant to the environment and poorly reflect the complex “wild” ecosystem, and organisms of interest may be so-far uncultured. Alternatively, one can model microbial populations using simple rules about their growth behavior and interaction patterns and thereby simulate dynamics of complex multispecies communities. Mathematical modeling has been used to explore processes such as biofilm formation (Wimpenny and Colasanti, 1997), response of the gut microbiota to changes in diet (Faith et al., 2011), and recovery of the microbial community after antibiotic treatment (Stein et al., 2013). Modeling allows one to investigate a range of situations and parameters and identify under what conditions robust inference can be made. Simulation therefore plays an important role in establishing a framework for exploring microbial diversity and identifying putative interactions in real ecosystems.

Here we use generalized Lotka-Volterra (gLV) modeling to simulate the dynamics of multispecies microbial consortia engaged in competitive and cooperative interactions. gLV models are based on simple rules about the effect of each species on each other. They have been applied as representations of microbial communities in diverse environments (Mounier et al., 2008; Rodriguez-Brito et al., 2010; Stein et al., 2013) as well as macro-ecological communities (Case, 2000). By simulating a range of conditions, we evaluate how well co-occurrence networks capture the underlying causal interaction structure by exploring their accuracy for mapping interactions. Investigated conditions cover sampling breadth (i.e., number of samples) and intrinsic ecological parameters such as diversity and interaction structure. We also identify topological features of the network that can be used to predict keystone species. Finally, we provide a summary of best practices and methodological considerations.

## Materials and methods

### Simulation of generalized lotka-volterra dynamics

Microbial community population dynamics were simulated using a generalized Lotka-Volterra model that follows the form:

$$\frac{dx_{i}}{dt}=r_{i}x_{i}\left(1-\frac{Ax}{k_{i}}\right)$$

where for any species *i* drawn from a meta-community, *x* is the vector of species abundances, *r*_*i*_ is growth rate of species *i*, *k*_*i*_ is the carrying capacity of species *i*, and *A* is a matrix containing interaction coefficients between species (Case, 2000). The model was evaluated using numerical integration with the *lsoda* function in the *R* package *deSolve* (Soetaert et al., 2010). Growth rates were assigned to each species from a uniform distribution from >0 to 1 so that all species were capable of positive growth. Carrying capacities were assigned to each species by drawing from either a β distribution, which allows one to simulate a range of distributions from a uniform distribution (with coefficients α = 1, β = 1) to an increasingly uneven distribution (e.g., α = 1 and β > 1), or alternatively from a lognormal distribution to simulate a very uneven distribution. Carrying capacity distributions were scaled to range between 1 and 100. Initial abundances of the species were drawn from a uniform distribution ranging between 10 and 100. The scaling of the carrying capacities and initial abundances was chosen for convenience and do not affect the outcomes of our investigation. The interaction matrix, which determines the topology of the metacommunity network, was assigned from different random models: (i) Erdös-Renyi model (Erdős and Rényi, 1959) resulting in a uniform distribution for interactions, (ii) the Watts-Strogatz model (Watts and Strogatz, 1998) forming networks with small world property, (iii) the Barabasi-Albert model (Barabasi and Albert, 1999) generating scale-free networks, and (iv) the Klemm-Eguiluz model (Klemm and Eguíluz, 2002; Prettejohn et al., 2011) generating topologies with small world and scale-free properties as well as modularity. Interaction magnitude was drawn from a uniform distribution between −1 and 1 (note that a negative value indicates synergy and a positive value antagonism) and the diagonal of the interaction matrix was set to unity to yield classic logistic growth dynamics in the absence of inter-specific interactions. The above-specified parameters were generated for a metacommunity that was subsequently subsampled to produce local communities. Subsampling was performed in a way to allow for a certain adjustable average percentage of shared species between local communities. The population dynamics of each local community was simulated using numerical integration until it reached steady state, when species abundances no longer changed with time, and the resulting species abundances were recorded. This process was repeated for the desired number of local communities.

### Co-occurrence networks

Co-occurrence networks were produced by applying an association metric or correlation coefficient to the simulated abundance data in a pair-wise manner. Statistically significant aggregation or avoidance was determined by generating a null distribution for each species pair by shuffling the site-abundance of one of the species and re-calculating the association metric. This resampling was performed 1000 times and the resulting distribution was used to generate *p*-values for observed association metric. *P*-values were corrected for multiple comparisons using the method of Benjamini and Hochberg (1995) and *p*-values less than *p* = 0.05 were considered to be statistically significant edges in the network. The *sparCC* program (Friedman and Alm, 2012), which was used for treatment of relative abundance data, uses a similar approach based on matrix permutation and null distribution generation.

### Evaluating network properties

To evaluate the topological properties of both the interaction and the co-occurrence network, we used the package *igraph* (Csardi and Nepusz, 2006) in the *R* environment. Particularly we were interested in properties potentially relevant for community roles and functioning as previously hypothesized in (Faust and Raes, 2012) and references therein, these are:

- Mean degree <k>: the degree of a node counts the number of edges it has. The mean degree is calculated over all nodes in the network.
- Degree distribution: the frequency of nodes vs. their (increasing) degree.
- Average shortest path length <l>: the shortest path between any two nodes is the single path with fewest edges between them. Alternative paths are feasible. The average shortest path length is the mean over all shortest paths between any two nodes in the network.
- Mean clustering coefficient <C>: a cluster of nodes is a triangle of nodes. The clustering coefficient calculates the fraction of observed vs. possible triangles for each node. The mean is subsequently determined from all nodes in the network.
- Betweenness centrality <CB>: the betweenness centrality of a node is equal to the number of shortest paths between any two nodes in the graph passing through that node. The mean is calculated from all nodes in the network.
- Closeness centrality <CC>: the closeness centrality of a node is given by the average distance of this node to any other node. Again, the network-wide measure is an average over all nodes in the network.

### Calculating sensitivity and specificity

The interaction network and co-occurrence network were compared to one another to determine the sensitivity and the specificity of the constructed co-occurrence network in detecting direct (first-order) interactions. For this calculation a true positive (TP) was indicated by the presence of an edge in the co-occurrence network that had the same sign as in the interaction network (when using association metrics with sign). A false positive (FP) was an edge in the co-occurrence network not present in the interaction network. A false negative (FN) was present in the interaction network but not the co-occurrence network. A true negative (TN) was not present in either interaction or co-occurrence network. Sensitivity was defined as TP/(TP+FN) and specificity was defined as TN/(TN+FP). In the case where two species interacted with each other with different signs, the interaction with the larger absolute value was taken to be the sign of the net interaction. Statistically significant differences were determined using analysis of variance and the Tukey method implemented in *R*.

### Keystone species analysis

Keystone species are commonly understood as species that have a disproportionate deleterious effect on the community upon their removal. The concept is straightforward if the community is pictured as interaction network with heterogeneous interaction patterns resembling dependencies within the community. We applied a brute-force leave-one-out strategy to evaluate the “degree of keystoneness” of a species in a given community network. We set the abundance of the species under investigation to zero and simulated community consolidation. The resulting steady state abundances were collected and compared to the original community abundances. We evaluated the impact of the keystone on species richness, in other words, the number of lost species in the steady state community. This procedure was applied to every node in the network to obtain a distribution of keystoneness, which was further used in correlations with topological measures. For co-occurrence networks we generated an average keystoneness for every node. The keystoneness of the node under investigation was evaluated for each different sampling site individually in the interaction network according to the procedure outlined above and subsequently averaged. Accordingly, the maximum keystone property of the interaction network constitutes the upper boundary for the mean keystone property of the co-occurrence networks.

Moreover we collected data about the interaction between the keystone and its target, particularly shortest path length and interaction type. The shortest path was used to record the depth of the keystone effect on the community in a standardized way. The interaction type is given in the interaction matrix, and we distinguished in a coarse-grained approximation antagonistic (−) from cooperative interactions (+). For long distance keystone interaction we used the product of all interactions along the shortest path between keystone and target. Keystones were classified by their topological properties using linear discriminant analysis (LDA) implemented in the *MASS* package in *R*. To do this, the dataset was randomly subsampled into two equally sized subsets with an equal number of keystones and non-keystones. An LDA model was constructed with one subset and the accuracy of the model was determined with the other subset. Accuracy was defined as number of correctly classified nodes divided by the total number of classified nodes.

## Results

### Generalized lotka-volterra models and co-occurrence networks

**Figure 1:**
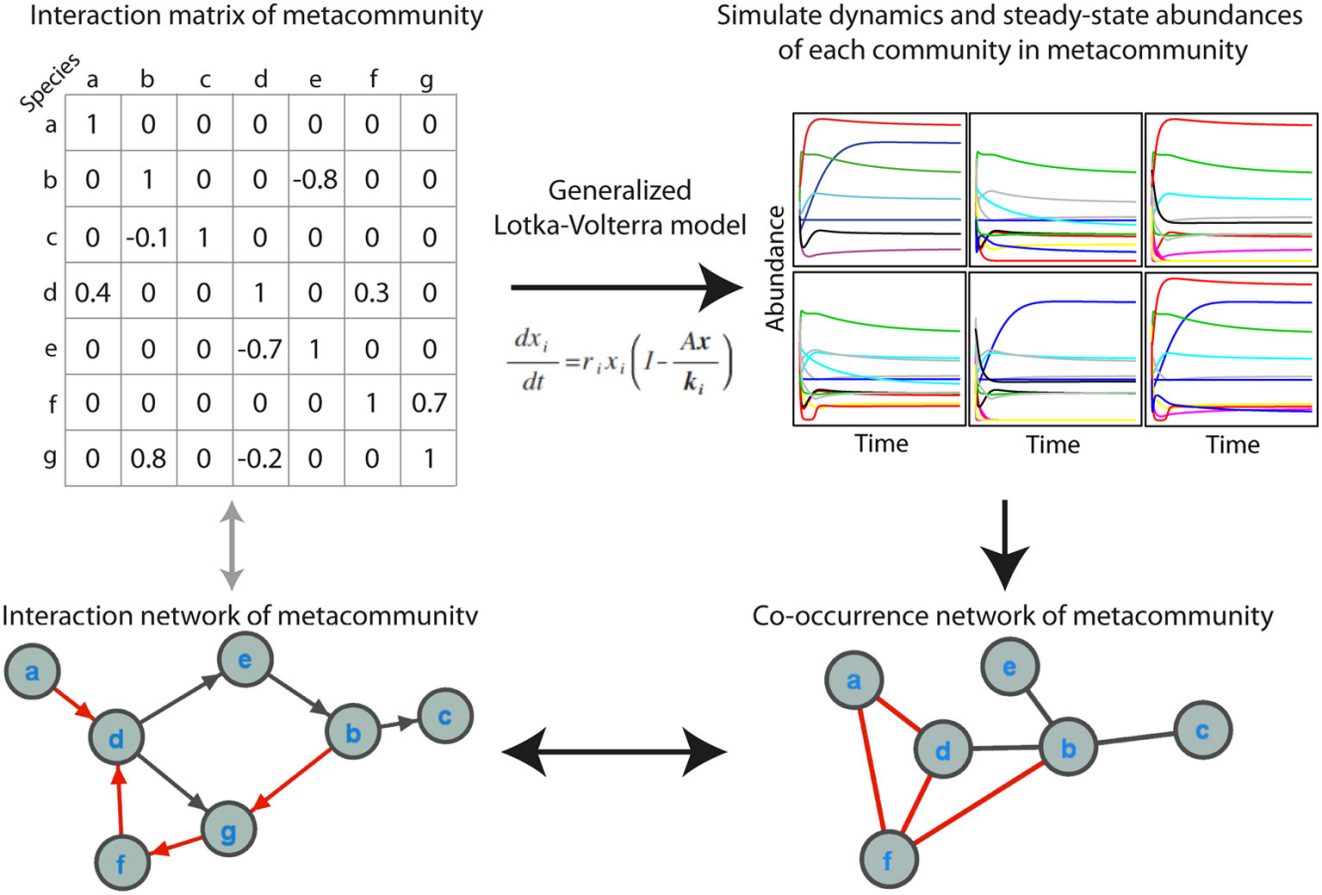
**Simulating microbial communities with generalized Lotka-Volterra modeling for co-occurrence network testing**. The main steps in the simulations are (1) producing an interaction matrix and directed network for a metacommunity, (2) simulating population dynamics in individual communities until steady state abundances are reached, (3) constructing a co-occurrence network, and (4) evaluating the extent to which the co-occurrence network reflects the interaction network, as well as the ecological significance of topological features of the network. Positive interactions and correlations are indicated in black and negative interactions and correlations in red. In the interaction network an arrow indicates the direction of interaction.

We simulated microbial metacommunities using generalized Lotka-Volterra (gLV) dynamics (Figure 1). Species subsampled from a metacommunity were used to produce local communities and community population dynamics were simulated until steady state abundances were reached. This process was repeated to produce a site-abundance matrix for the species in each metacommunity, which was then used to build a co-occurrence network using a variety of standard methods. As described below, we explored the effects of a range of experimental and ecological parameters on co-occurrence network performance. When not otherwise specified we used a “standard community” with an average 100 species per site, carrying capacities drawn from a uniform distribution, 80% shared species between sites, a random interaction network structure with an average of 2 interactions per species, and a “standard network” constructed from 100 sampling sites with absolute abundance data and the Spearman correlation coefficient.

### Experimental parameters

**Figure 2:**
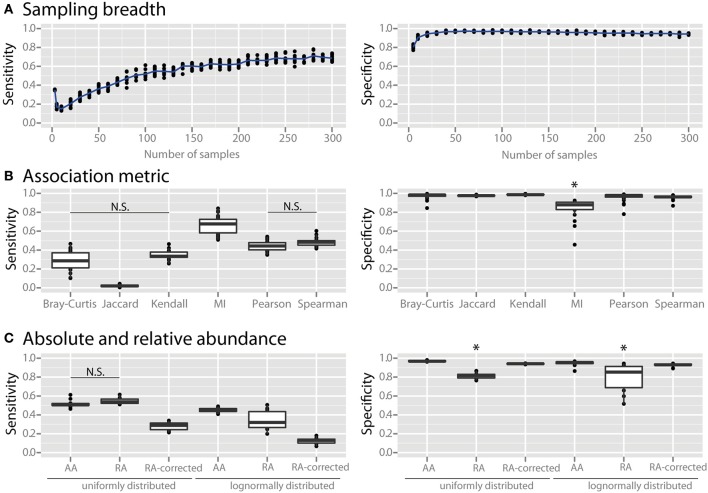
**Effect of experimental and analytical parameters on co-occurrence network performance**. The sensitivity and specificity of co-occurrence networks in revealing direct interactions was tested using the standard community of 100 species per site, 100 sites with 80% species overlap between them, carrying capacities drawn from a uniform distribution and an average of 2 interactions per species, while varying the following parameters: **(A)** sampling breadth (i.e., number of samples), **(B)** association metric used (MI = mutual information score), **(C)** and use of absolute abundance (AA), relative abundance (RA), or *sparCC*-corrected relative abundance (RA-corrected) data. For **(C)** the different data types were compared for communities with uniformly- or log-normally-distributed species abundances. All comparisons in the left panel of **(B,C)** are significant (*p* < 0.05) except where denotes with N.S. ^*^indicates *p* < 0.05 for all comparisons against all other conditions that are not starred.

We examined the effects of three major factors that can be determined by the experimenter: (1) the number of sampling sites, (2) the association metric applied, and (3) the use of absolute vs. relative abundance data. When using only a small number of samples the specificity of co-occurrence networks was low, but it increased with an increasing number of sites until it plateaued at about 25 sites (Figure 2A). As the number of sites was further increased the sensitivity continued to increase, although it also appeared to slowly plateau. We compared several commonly used association metrics: Bray-Curtis dissimilarity, Jaccard index, mutual information score, Kendall coefficient, Pearson coefficient, and Spearman coefficient. All metrics yielded highly specific networks except for mutual information score, which had significantly lower specificity (Figure 2B, all comparisons are significant with a *p* < 0.05). Among the metrics with high specificity, Pearson and Spearman coefficients had the highest sensitivity, followed by Kendall coefficient and Bray-Curtis dissimilarity, and then Jaccard index (*p* < 0.05 for all comparisons except Pearson vs. Spearman and Kendall vs. Bray-Curtis). We compared co-occurrence network performance from communities with either uniform or log-normal distributed species when constructed with absolute data, relative abundance data, and sparCC-corrected relative abundance data. Network specificity was reduced for relative abundance data but was restored by SparCC correction (*p* < 0.05 for both uniform and lognormal distributions). However, SparCC correction led to a decrease in sensitivity compared to absolute abundance data (Figure 2C, *p* < 0.05 for both uniform and log-normal distributions).

### Ecological parameters

**Figure 3:**
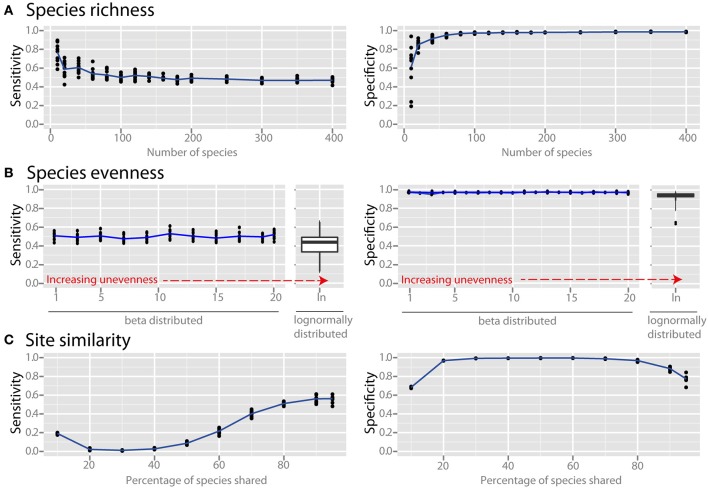
**Effect of ecological properties on co-occurrence network performance**. The sensitivity and specificity of co-occurrence networks in revealing direct interactions was tested using the standard community, while varying aspects of α and β diversity. Properties evaluated were the diversity properties: **(A)** per-site species richness, and **(B)** species evenness. Evenness was controlled by drawing species carrying capacities from either (i) a scaled β distribution (α parameter = 1) and tuning β from 1 (equivalent to a uniform distribution) to 20 to increase unevenness, or (ii) a scaled lognormal distribution. **(C)** β diversity was evaluated by modifying the site similarity (or Jaccard similarity), which is the mean percentage of shared species between any two local communities in the meta-community.

**Figure 4:**
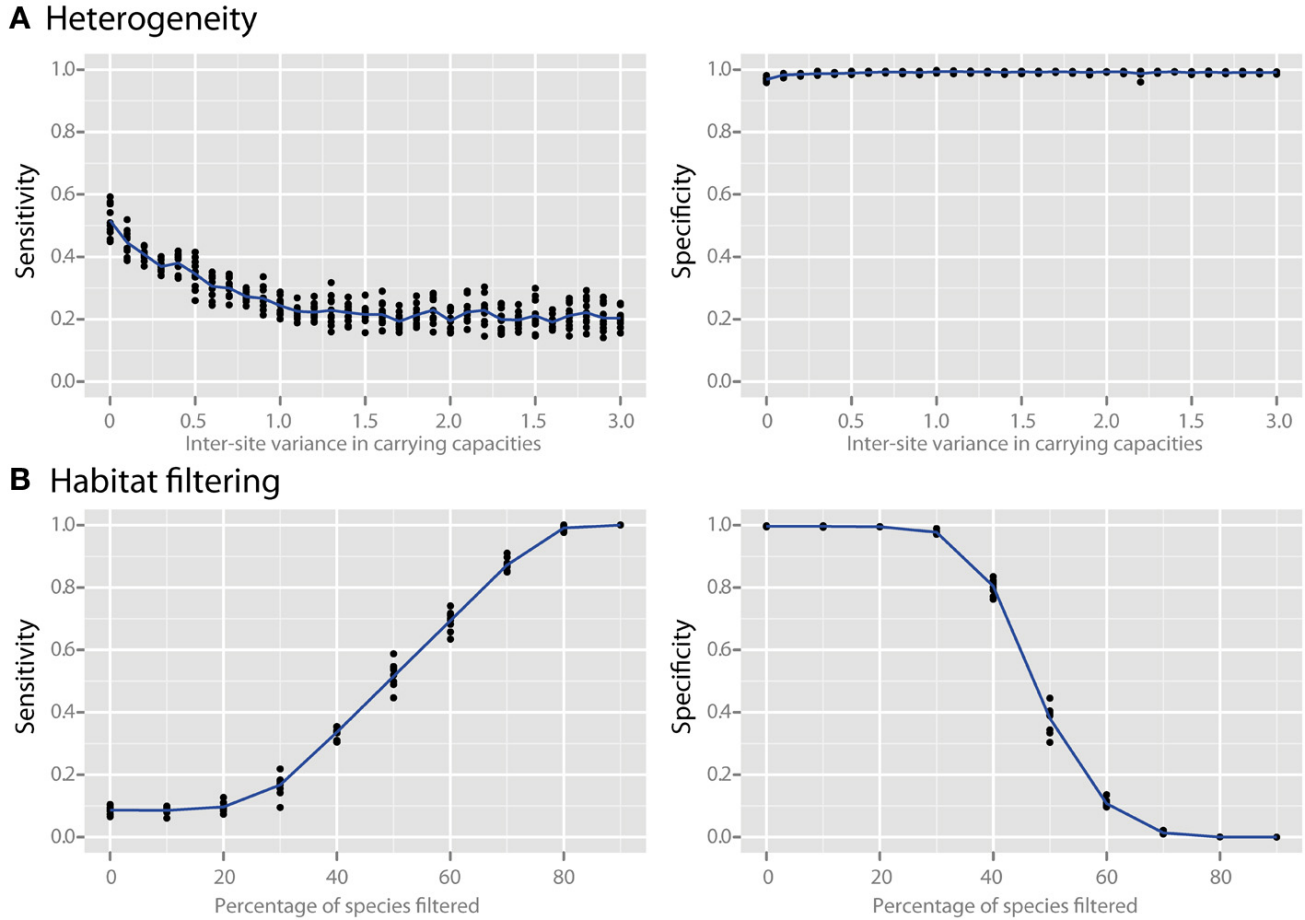
**Effect of heterogeneity and habitat filtering on co-occurrence network performance**. The sensitivity and specificity of co-occurrence networks in revealing direct interactions was tested using the standard community, while varying carrying capacity and species overlap. **(A)** Heterogeneity was simulated by stochastically varying species carrying capacities at each local site with a certain variance. This is analogous to the additional noise that would be expected if sites were near to, but not yet in, steady state. **(B)** The effect of habitat filtering was explored as a function of filtering intensity, which is the percent of the metacommunity that cannot occupy multiple habitats (i.e., percent habitat specialists).

Species richness and species evenness generally did not influence network sensitivity or specificity (Figures 3A,B, respectively), though there was a dramatic loss of specificity for very low species richness (10–20 species). Beta diversity, which was calculated using Jaccard similarity, had a large impact on network sensitivity. Specificity remained high at Jaccard similarities ranging from 20 to 80%, but sensitivity increased with increasing similarity. To evaluate the effect of site heterogeneity on network inference, we randomly varied the carrying capacities of each species at each site. With increasing heterogeneity the co-occurrence network sensitivity initially dropped rapidly and then plateaued, but the specificity remained high (Figure 4A). We then considered the case of exclusionary environments in which only a subset of species can survive in both habitats. We simulated two habitats to which a certain proportion of the species in the metacommunity were exclusively associated while keeping constant the within-habitat site similarity. When more than 20–30% of species were exclusive to only one environment the specificity of the networks rapidly declined (Figure 4B).

**Figure 5:**
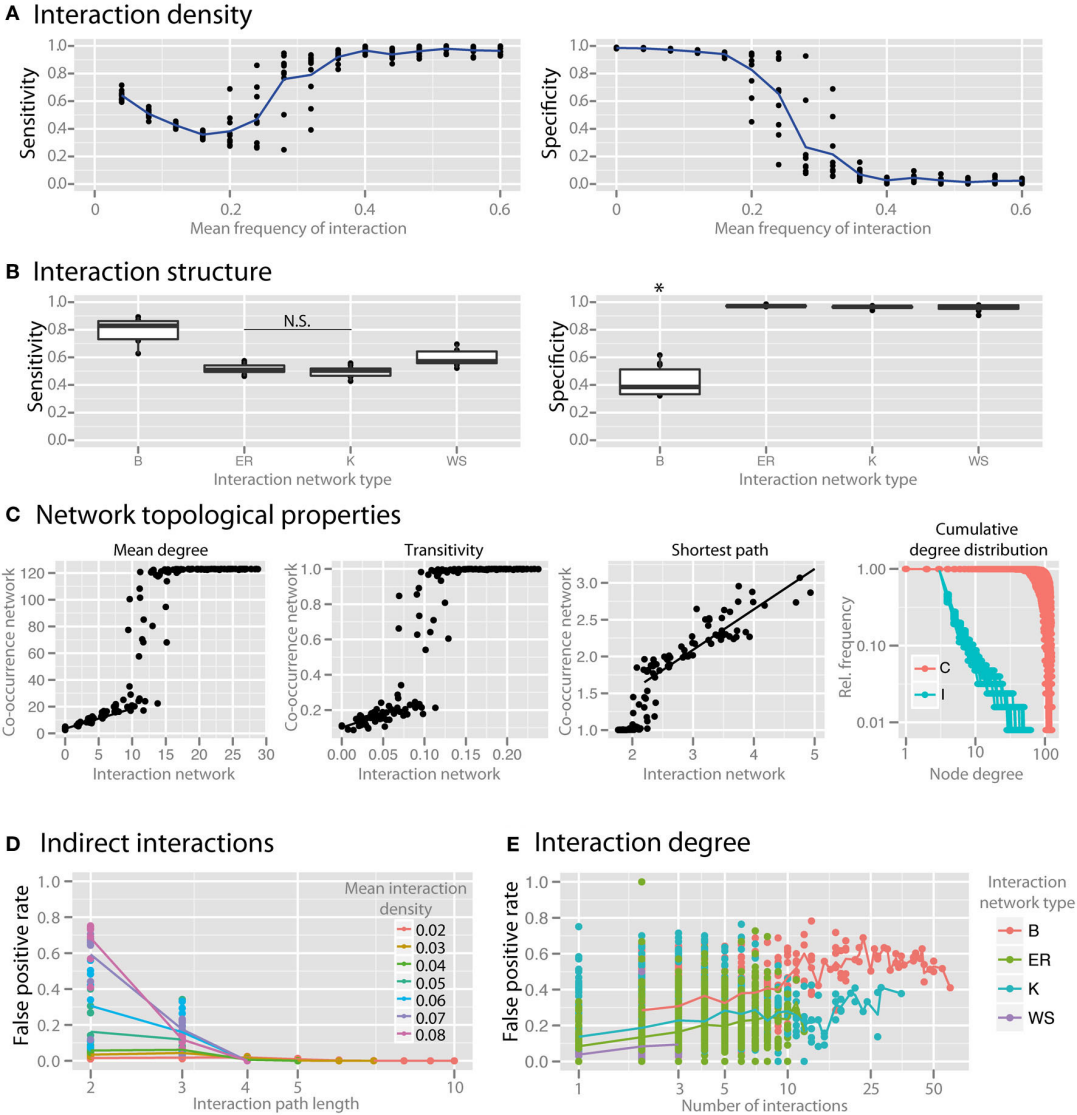
**Effect of interaction structure on co-occurrence network performance**. The sensitivity and specificity of co-occurrence networks in revealing direct interactions was tested using the standard community under different interaction scenarios. **(A)** Interaction density of random (ER) networks, or the mean frequency of an interaction between any two species, was varied between 0 and 0.6. **(B)** Interaction networks with different structures but with the same mean interaction density (0.02) were simulated. Interaction networks were chosen to have random (ER), small-world (WS), scale-free (B), and small-world, scale free and modular (K) properties. **(C)** The ability of the co-occurrence network to reproduce the interaction network topology was examined for a few key network parameters: the mean degree, transitivity, the mean shortest path length, and cumulative degree distribution. Black lines indicate regions for which a linear model was fit. Standard community with mean species number set to 50 per site was used for **(C–E)**. **(D)** For ER networks of varying interaction densities, the false positive rate (FPR) was determined with respect to the interaction path length between the species incorrectly identified as directly interacting. **(E)** For different interaction network structures, the per-species FPR was identified with respect to the number of interactions (i.e., the interaction degree) of the species. All comparisons in the left panel of **(B)** are significant (*p* < 0.05) except where denotes with N.S. ^*^indicates *p* < 0.05 for all comparisons against all other conditions that are not starred.

Interaction density (i.e., the average number of interactions per species) dramatically reduced the specificity of co-occurrence networks (Figure 5A). To examine the impact of the topological interaction structure on co-occurrence inference, we compared random networks (Erdös-Renyi, ER), small-world networks (Watts-Strogatz, WS), scale-free networks (Barabasi-Albert, BA), and small-world, scale-free networks with some modularity (Klemm-Eguiluz, K). Barabasi-Albert scale-free networks had the lowest specificity (Figure 5B, *p* < 0.05). Key topological properties of the network were reproduced at lower but not higher interaction density (Figure 5C, mean degree: *r*^2^ = 0.81 between 1 and 10, transitivity: *r*^2^ = 0.42 between 0 and 0.05, shortest path: *r*^2^ = 0.77 when >2). Centrality measures (betweenness and closeness centrality) were not well reproduced (*r*^2^ = 0.24 and 0.11, respectively). The power law distribution of scale-free networks was also not well reproduced (Figure 5D). We examined the false positive rate (i.e., rate of spurious correlations) as a function of interaction path length and found that in random networks the FPs were primarily driven by species connected by a path of less than or equal to 3 (Figure 5D). The false positive rate (with interaction path length 2) was higher with higher node degree (Figure 5E).

### Keystone species

**Figure 6:**
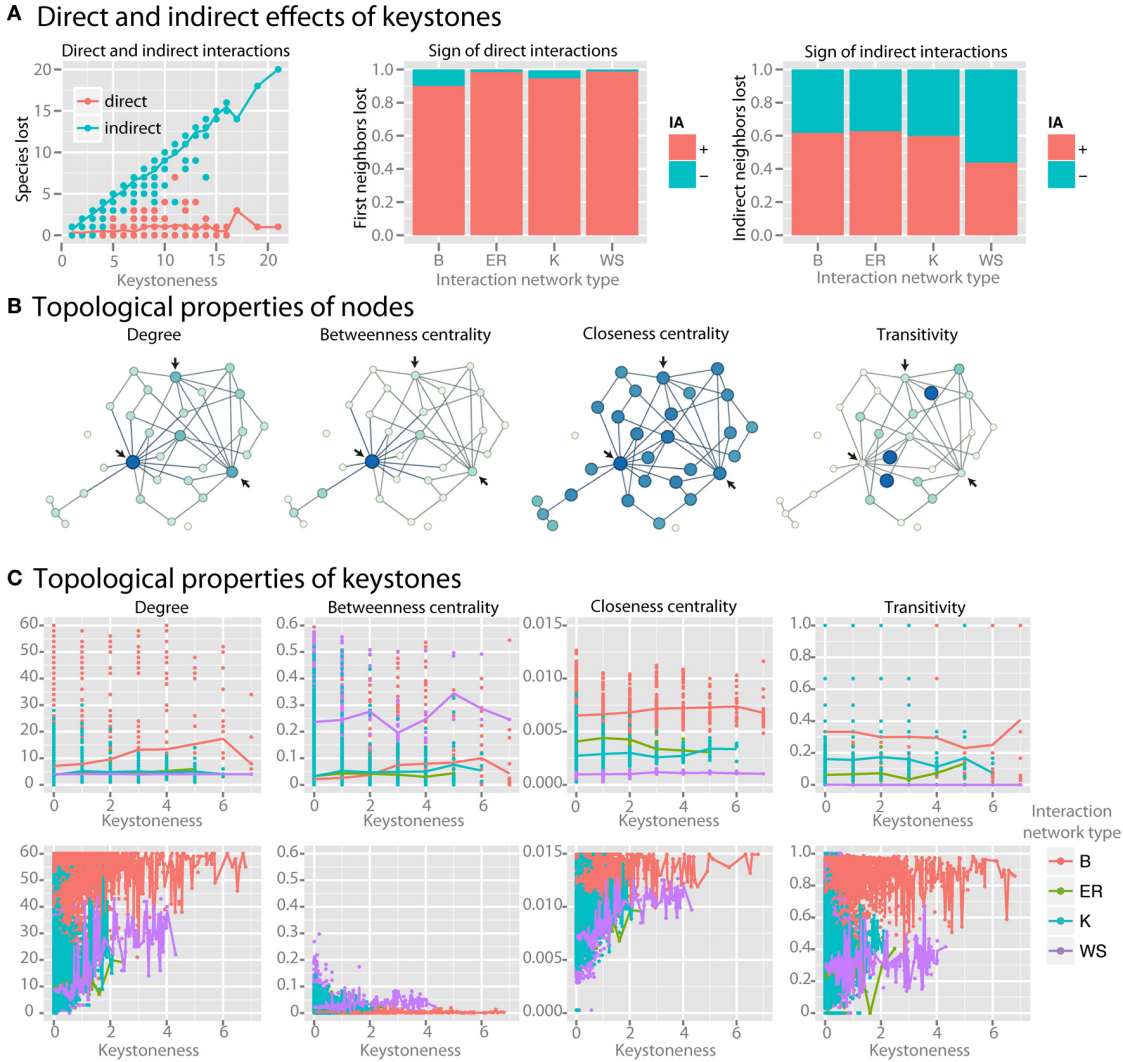
**Identifying keystone species in co-occurrence networks**. For keystone species analysis standard communities with mean species number of 50 species per site were used. **(A)** For each species, the number of species lost when it is removed from the community is plotted. The larger number of species lost, the higher the keystoneness. Lost species are separated into those that interacted either directly or indirectly with the keystone, and the sign (for direct interactions) or the net sign (for indirect interactions) of the interaction is shown. **(B)** Selected topological properties are shown in example networks, with the color (from light to dark) and size (from small to large) of each node scaled to the value of the property. Arrows indicate possible keystone species based on the results shown in **(C)**. **(C)** Topological properties of keystones in both the interaction network (top row) and the co-occurrence network (bottom row) colored by interaction network type are shown.

As keystoneness of a species increases, the number of direct interactions that it engages in does not increase, but the number of species that are affected indirectly by it increases linearly (*r*^2^ = 0.94) (Figure 6A). Species directly affected by the loss of a keystone had positive interactions with the keystone (Figure 6A). Species indirectly affected by keystones, however, had a roughly equal number of net positive and negative interactions with the keystone species along the most direct path via common neighbors. We examined whether topological properties in interaction or co-occurrence networks could be used to identify keystone species (examples of selected parameters are shown in Figure 6B). We found no strong patterns for keystone species in four major topological parameters in the interaction network (Figure 6C). Co-occurrence networks, however, did show trends, with keystone species tending to have high mean degree, low betweenness centrality, high closeness centrality, and high transitivity (Figure 6C). By employing these four topological features in a LDA model were able to correctly classify nodes as keystones with 85% accuracy even when a relatively low level of keystoneness (≥2) was defined as a threshold. For higher keystoneness thresholds the accuracy could be further improved.

## Discussion

### Simulation of complex microbial communities

Microbial ecosystems can harbor a tremendous phylogenetic and functional diversity of organisms engaged in manifold activities and embedded in an unseen network of interactions. Understanding recurrent patterns of microbial community organization, community roles and detailed dependencies between species requires causal evidence of these microbial interactions. But, given the complexity of most microbial habitats, we are currently heavily limited in our attempt for direct insight. In an alternative approach, co-occurrence networks are produced using correlation of abundance patterns from gene-targeted (e.g., 16S rRNA gene) or metagenomic sequencing data. These correlation networks have been proposed as a means to approximate microbial interactions, but validation is generally lacking. In order to evaluate the performance of co-occurrence networks, we simulated microbial metacommunities of species that can interact with one another using generalized Lotka-Volterra (gLV) dynamics (Figure 1). The term species is used here as a general term for a biologically meaningful unit, which could also be substituted with other terms such as “operational taxonomic unit,” strain or serotype. The beauty of this approach is that here, unlike in nature, the real interactions and interaction structure in the metacommunity is known. By comparing the network of interactions to the co-occurrence network we can therefore directly judge the quality of the co-occurrence network in revealing the underlying interaction structure.

### Effects of sampling breadth and analysis options

Irrespective of the microbial ecosystem under investigation, a researcher must decide how to best sample for and analyze microbial survey data. We examined the effects of sample number, the association metric used, and absolute vs. relative abundance data. As expected, we observed a loss of specificity in the co-occurrence network when using only a small number of samples applied to absolute abundance data (Figure 2A). The specificity of the network increased with an increasing number of sites until it plateaued at about 25 sites. Sensitivity could be improved further using up to 100 sites. This indicates that networks constructed with a low number of sites are susceptible to FP edges and should therefore be interpreted with caution.

We next evaluated the effect of association metric on the performance of the co-occurrence network by applying several commonly used metrics (Figure 2B). All tested metrics yielded highly specific networks except for mutual information score. Mutual information score also does not include the sign of interaction (i.e., whether an interaction is positive or negative). We therefore suggest that mutual information score be used only in combination with a second metric in order to give the interaction a sign and to increase specificity. For the other examined metrics, Pearson and Spearman coefficients had the highest sensitivity (Figure 2B). The Jaccard index, which is a presence-absence metric, had very low sensitivity compared to the other metrics. This indicates that quantitative species abundance, and not just presence or absence, contains important information about species-species interactions. Association metrics are used to compare the similarity of microbial communities in a variety of applications and it is known that metrics differ in their performance in detecting different patterns (Lozupone et al., 2007). In our analysis Spearman and Pearson correlation coefficients were the top performers for detecting interactions in metacommunities from relatively similar communities and absolute abundance data, but other metrics may prove to be superior in other applications.

Almost all sequencing surveys employ relative abundance measures rather than quantifying absolute abundances of microorganisms. This is known to lead to a bias in which spurious correlations are produced, particularly when the community is uneven and has low species richness (Friedman and Alm). There are two options open to the investigator: either to quantify total absolute abundances of all bacteria—using, for example, qPCR to determine total bacterial gene copy numbers in each sample—in order to convert relative compositional data to absolute data, or to employ a correction using the principle of sub-compositional coherence (Aitchison, 2003) before correlation analysis, such as is done in the program *sparCC* (Friedman and Alm, 2012). As expected, the specificity of networks suffered when relative abundance data was used, and sparCC correction was able to eliminate spurious correlations (Figure 2C). Interestingly, sparCC correction also reduced the sensitivity of these networks compared to absolute abundance data. While absolute abundance data is the gold standard for network construction, we recognize that it is not always feasible to produce this data. For cases in which only relative abundance data is available *SparCC* correction is a valuable tool, but it should be kept in mind that sensitivity might be lost when applying this correction.

### Effect of alpha and beta diversity

Microbial communities in different environments can vary widely in their composition and structure. Though the experimenter cannot necessarily influence ecological parameters, it is valuable to know which factors may cause problems in co-occurrence network inference. We considered the effect of species richness, community evenness, and similarity of communities across sampling sites. Species richness and species evenness, which are aspects of what is known as α (or within-site) diversity, did not have a large influence on network sensitivity and specificity (Figures 3A,B), though at very low species richness (10–20 species) there was a dramatic loss of specificity. This was not an artifact of relative abundance data since absolute abundance was used, but rather likely arises from the relatively interaction-rich nature of these low species richness communities (discussed in the section below). Most environments are relatively species-rich, with estimates of about 10^2^–10^4^ species (Fierer and Lennon, 2011), but network inference could be problematic for low-species-richness environments such as the atmosphere (Bowers et al., 2009), acidic environments (Baker and Banfield, 2003; Tyson et al., 2004), and glacial ice (Simon et al., 2009) if species that are present in these environments are interacting frequently with one another. The structure of most microbial communities is known to be extremely uneven, with a few dominant taxa overshadowing many rare taxa (Huber et al., 2007; Bent and Forney, 2008). Our analysis suggests that community evenness does not directly affect co-occurrence network sensitivity and specificity. However, it may have an indirect effect because uneven communities require increased sampling depth in order to detect the real species richness, and if this is inadequate the number of detected species (i.e., the effective richness) will be reduced.

The diversity of communities between different sites, or β diversity, can be calculated via a variety of metrics (Lozupone et al., 2007). We used a simple and intuitive metric to quantify the similarity of communities at different sampling sites: the average percentage of species shared between any two sites (i.e., the Jaccard similarity). The similarity of communities had a large effect on network sensitivity (Figure 3C). Though specificity remained high at similarities ranging from 20 to 80%, the sensitivity increased through this range with increasing similarity. Samples with relatively high similarity in species membership are therefore useful for constructing sensitive networks. Many real microbial communities have a lower percentage of shared taxa (e.g., Tap et al., 2009; Zinger et al., 2011), but this is largely due to undersampling of rare species (Lemos et al., 2012). In this case, we recommend removing species that are present in less than 20% of the sampling sites to avoid spurious correlations. Specificity also suffered for communities with average shared species above 90%, but this will likely not be a problem in practice because this level of similarity is seldom encountered in microbial surveys.

### Effect of heterogeneity and gradients

The communities evaluated thus far have been at steady state, a simplification that may not always be representative of many complex communities (Briones and Raskin, 2003; Curtis and Sloan, 2004; Shade et al., 2012). We therefore looked at how variability in properties of local sites affects network inference. This was done by randomly varying the carrying capacities of each species at each site, which can be interpreted as adding inter-site heterogeneity in which each species has a greater or lesser advantage, and thereby essentially adds “noise” to the dataset. We discovered that as the heterogeneity was increased the co-occurrence network sensitivity initially dropped rapidly and then plateaued, but unexpectedly the specificity remained high (Figure 4A). This indicates that co-occurrence networks are robust to small differences in sample sites or near-steady-state community conditions, and that while some sensitivity is lost the specificity of the resulting networks remains reliable.

Microbial surveys commonly compare samples across environmental gradients (such as pH or temperature) or in very different habitats (e.g., freshwater vs. saltwater) in which different species are expected to thrive, which is also called habitat filtering (e.g., Caporaso et al., 2011). To examine how co-occurrence networks perform when sampling gradients or between environments in which only a subset of species can survive in both habitats, we evaluated the case of two habitats exclusive to a certain proportion of the species (habitat specialists) in the metacommunity, while keeping constant the within-habitat site similarity (Figure 4B). We found that when more than 20-30% of species were habitat specialists and survived in only one environment, the specificity of the networks rapidly declined. This loss in specificity occurs because the co-occurrence network is unable to distinguish whether a statistically significant co-occurrence is due to an interaction or rather to a shared habitat preference. In other words, species that aggregate in certain sites due to environmental factors but do not interact will appear in co-occurrence networks along with species that are habitat generalists but that truly interact with one another. This very important result underscores that when co-occurrence networks are used to infer putative interactions samples should be drawn from similar environments in order to minimize the effects of habitat filtering or else the resulting network will suffer from a lack of interpretability.

### Effect of interaction density and structure

While various mechanisms of cooperative and competitive interactions have been identified, little is known about the prevalence and importance of these in natural communities (Hibbing et al., 2010). *In vitro* and *in silico* analyses have demonstrated widespread competition (Foster and Bell, 2012) as well as unexpected cooperation with increasing species richness (Freilich et al., 2011). In our simulations we found that interaction density—the probability that there is an interaction between any two species—has a dramatic effect on the specificity of co-occurrence networks (Figure 5A). As interaction density increases the specificity of the network is lost, which is due to higher-order correlation in a denser network (i.e., the situation where two species do not interact with each other but both interact with a third species and as a result are correlated due to an indirect interaction) (Krumsiek et al., 2011). This effect also explains why low richness communities in the simulations discussed above had low specificity: when evaluating species richness the average number of interactions per species was kept constant and therefore the interaction density increased as species richness decreased (Figure 3A). Note however that this is not a mere theoretical problem but can be easily encountered in communities of strongly interacting microbial clusters (Tyson et al., 2004; Tringe et al., 2005).

To step beyond interaction density, we considered the impact of the topological interaction structure on co-occurrence inference. Alternative topological patterns of community-wide interactions can enforce or hinder robustness and resilience of the community and are therefore important for community productivity. Interaction structure in complex microbial communities is unknown, so we utilized network models with different properties to consider the possible parameter space. We investigated to what extent the particular structure of interactions influences the community representation in the co-occurrence network. Recurrent topological properties observed in macro-ecological interaction networks include small-worldness, scale-freeness and modularity (Montoya et al., 2006; Thébault and Fontaine, 2010). Here we used standard network algorithms generating random networks with the outlined topologies, i.e., respectively Watts-Strogatz, Barabasi Alberts, and Klemm-Eguiluz, the latter of which features all three properties and might thus be the closest, yet very coarse-grained approximation to complex microbial communities. We found that BA scale-free networks were the most susceptible to spurious correlations (Figure 5B). BA networks tend to have species that engage in many interactions. These hub species interact with many other species and by that increase the possibility of wrong correlations between species indirectly connected by a hub (Krumsiek et al., 2011). Similarly, when comparing the ability of the co-occurrence network to reproduce the topological properties of the interaction network, we found that key properties such as mean degree, transitivity, and mean shortest path length are reproduced well at lower interaction density, but not as interaction density increases (Figure 5C). Also, the power law distribution representative of scale-free networks was scarcely reproduced (Figure 5D), indicating that scale-free interaction patterns in microbial communities may not be well represented by the degree distribution of co-occurrence networks.

In order to further evaluate how spurious correlation is distributed in the network, we looked at the false positive rate with respect to the distance between species in the interaction network. For example, species that directly interact with each other have a minimal path length of 1, and species that do not directly interact with each other, but both interact with a common third species would have a minimal path length of 2. Spurious correlations cannot, by definition, occur when species have an interaction path length of 1 (since these are interacting species), so we examined the rate of FP correlations in path lengths >1. We found that in random networks with different interaction densities most spurious correlations were found between species with path length 2 or 3 (Figure 5D), which strongly suggests that co-occurrence network specificity can be compromised when indirect effects from interacting species in a complex community come into play (Krumsiek et al., 2011). We revisited the false positive rate between species connected via a common first neighbor (shortest path = 2) vs. their node degree (number of interactions) across network topologies (Figure 5E). We found that highly interacting hub species, which were most prevalent in BA networks, are responsible for the highest number of false correlations. Network hubs, therefore, are hot spots for spurious correlations due to the local density of interactions. Future work is needed to develop a procedure to correct for this problem and to control for the false positive rate in interaction-rich networks and in neighborhoods of hubs.

### Keystone species in networks

Keystone species are commonly defined as species that exert a disproportionately large effect on the ecosystem relative to their abundance (Power et al., 1996). In macro-ecology top predators are often considered keystone species because they control the population sizes of prey species, but examples from microbial ecosystems include low abundant but highly active sulfate reducers that mediate a major biogeochemical process (Pester et al., 2010) and primary degraders of recalcitrant substrates in the gut that make available substrates for other organisms (Ze et al., 2012). Collectively, the impact these keystones have on their communities is shaped by the repertoire of interactions with other members. In terms of interaction topology we hypothesized that keystone behavior leads to recurrent topological patterns in the network.

We searched for keystone species in both the interaction network and co-occurrence network by reasoning that keystone species should have a large impact on the community composition, and therefore that absence of the keystone species should lead to major losses in community members. We found that as a species in the native interaction network becomes more and more of a keystone, the number of direct interactions that it engages in does not increase. Instead, the number of species that are affected indirectly by the keystone, i.e., via one or more community members, does increase linearly (*r*^2^ = 0.94) (Figure 6A). As expected species directly affected by the loss of a keystone strongly tended to have positive, or synergistic, interactions with the keystone (Figure 6A). Interestingly, species indirectly affected by keystones had a roughly equal number of net positive and negative interactions with the keystone species along the most direct path via common neighbors, where negative net interactions between keystone and affected species are more pronounced in better-linked network topologies with increasing small-world property. Thus their loss from the community is likely due to the increasing number of alternative interaction paths connecting them with keystone species (Figure 6A).

We then asked whether we could identify keystone species via topological properties in interaction or co-occurrence networks (examples of selected parameters are shown in Figure 6B). We found no strong patterns for keystone species in four major topological parameters in the interaction network across four particular network shapes (Figure 6C). Co-occurrence networks, however, did show trends, with keystone species tending to have high mean degree, low betweenness centrality, and high closeness centrality (Figure 6C). Some species with low keystoneness also shared these properties, so even if these properties seem to be prerequisite for keystones, they are not highly specific. However, these properties could be used to classify nodes as keystones with at least 85% accuracy in a LDA model, indicating that they do have predictive power. This analysis reveals that keystone species are more detectable in co-occurrence networks than interaction networks with the tested topological shapes, and that keystones in co-occurrence networks tend to be highly connected and centrally-clustering nodes. More generally, keystoneness is a result of interaction topology. Depending on the interaction topology and type (beneficial/antagonistic), keystone species can promote or reduce species richness. Therefore, we would expect that no matter whether keystones are defined as species that have either a relatively large positive or negative impact, they would be identifiable by the same topological features in the co-occurrence networks.

### Methodological considerations

In the above analysis we have assumed that the input data is able to quantitatively represent all species (or any other biologically meaningful unit) present in an environment. There are several methodological problems that may violate these assumptions in 16S rRNA-based (or other gene-targeted) sequencing surveys. Primers for gene-targeted surveys are designed to maximize coverage (Klindworth et al., 2013) and therefore commonly include degeneracies that can lead to biased amplification in multi-template PCR (Chandler et al., 1997) and that the original ratio of templates is not preserved after PCR (Polz and Cavanaugh, 1998). Additionally, library preparation for next-generation sequencing using sequencing-adapter- and barcode-containing primers can introduce biases (Berry et al., 2011). It is therefore advisable to use a low-cycle number PCR, pool replicate PCR reactions, and use a two-step PCR protocol for preparing barcoded sequencing libraries (Polz and Cavanaugh, 1998; Berry et al., 2011).

In addition to technical biases, biological processes can also lead to violations in our assumptions. Microorganisms have variable copy number of targeted genes such as rRNA genes (Farrelly et al., 1995), which can influence abundance in sequencing libraries. In addition genomes with several gene copies may have non-identical copies and this intragenomic heterogeneity (Acinas et al., 2004; Sun et al., 2013) can lead to counting one species as several species. Alternatively, the targeted gene or gene region may have insufficient resolution to resolve different species, as is the case for the 16S rRNA gene in the *Escherichia coli* (Wirth et al., 2006), or ecologically meaningful taxa. While some functions are well conserved in lineages, such as for example photosynthesis, the phylogenetic signal of many functions is lost extremely rapidly, with organisms more than 1% divergent in the 16S rRNA gene having completely different carbohydrate utilization profiles (Martiny et al., 2013). Finally, and most challenging to study or to evaluate, microorganisms may have different activities in different local communities. Phenotypically plastic organisms could engage in different metabolisms and even have different interactions under different conditions (Klitgord and Segrè, 2010; Berry et al., 2013). In order to minimize the above-listed problems, we recommend to use the highest level of sequence resolution possible with the targeted gene and sequencing approach, to evaluate if any taxa are covarying to such a great extent that it is likely that the targeted genes are chromosomally-linked (Sunagawa et al., 2013), and to compare relatively similar environments in which it is less likely that organisms drastically change their metabolisms or interaction patterns.

Based on our findings, we have developed a set of best practices to guide co-occurrence network analysis (Box 1).

Box 1 **Best Practices for Co-Occurrence Network Construction and Inference**

- Filter out infrequent species. Remove infrequent species from dataset until mean site similarity (Jaccard similarity) is at least 20%. Communities with very low similarity produce less specific co-occurrence networks. Removal of additional species will increase the sensitivity of the network.
- Sequence communities with highly uneven composition more deeply. This is necessary to achieve a high coverage and to therefore recover most or all potentially interacting species.
- Include as many samples as possible. Co-occurrence networks produced from few samples are highly unreliable. We recommend using a minimum of 25 samples, but including even more samples will increase sensitivity for co-occurrence events.
- Include only samples from similar environments. Samples from very different environments are likely to subject species to habitat filtering, which makes network interpretation impossible. Co-occurrence networks are robust to some heterogeneity in environments, but the less heterogeneity is included the more robust and sensitive will be the network. Outlier samples (e.g., a seawater sample in a freshwater survey) should be removed before network construction.
- Use absolute abundance, or *sparCC*-corrected relative abundance data. Relative abundance data suffers from apparent correlations, which reduces the specificity of the network. This can be avoided by using absolute abundance data or by applying a correction to relative abundance data using the principle of subcompositional coherence.
- Use sequencing data at the highest resolution possible. Clustering of data into operational taxonomic units or “species” should be done at the maximum possible sequence similarity (given the capabilities and error rates of the sequencing technology used) in order to avoid grouping unlike organisms. Oppositely, groups with high sequence similarity that correlate extremely highly should be closely examined to ensure that they are not two gene copies on the same chromosome.
- For absolute abundance data, use Spearman or Pearson correlation coefficients to construct co-occurrence networks. These two coefficients outperform other tested metrics in their sensitivity and specificity for detecting interactions in absolute abundance data.
- Be aware of spurious correlation due to indirect interactions. Interaction dense communities and species with a high number of correlations tend to have increased rate of spurious correlations, so be particularly cautious of inferring a direct interaction between any two species in the neighborhood of hub or highly-connected species.

## Conclusion

Using a simulation approach we have shown that co-occurrence networks can indeed identify putative interactions between microorganisms in the environment, but that the performance of networks is highly dependent on several factors. Experimental design and analysis options must be carefully considered in order to produce co-occurrence networks that can be reliably interpreted, and to facilitate this we outline key best practices to follow. When properly produced, co-occurrence networks can reveal possible community interaction patterns and are therefore a powerful tool for generating hypotheses about interactions that can then be tested in targeted experiments.

## Author contributions

David Berry and Stefanie Widder both significantly contributed to the design and interpretation of the work as well as paper-writing.

### Conflict of interest statement

The authors declare that the research was conducted in the absence of any commercial or financial relationships that could be construed as a potential conflict of interest.

## References

[B64] Acinas S. G. Marcelino L. A. Klepac-Ceraj V. Polz M. F. (2004). Divergence and redundancy of 16S rRNA sequences in genomes with multiple rrn operons. J. Bacteriol. 186, 2629–2635 10.1128/JB.186.9.2629-2635.2004 15090503 PMC387781

[B1] Aitchison J. (2003). A concise guide to compositional data analysis, in 2nd Compositional Data Analysis Workshop (Girona).

[B2] Baker B. J. Banfield J. F. (2003). Microbial communities in acid mine drainage. FEMS Microbiol. Rev. 44, 139–152 10.1016/S0168-6496(03)00028-X 19719632

[B3] Barabasi A. Albert R. (1999). Emergence of scaling in random networks. Science 286, 509–512 10.1126/science.286.5439.509 10521342

[B4] Barberán A. Bates S. T. Casamayor E. O. Fierer N. (2012). Using network analysis to explore co-occurrence patterns in soil microbial communities. ISME J. 6, 343–351 10.1038/ismej.2011.119 21900968 PMC3260507

[B59] Benjamini Y. Hochberg Y. (1995). Controlling the false discovery rate: a practical and powerful approach to multiple testing. J. R. Stat. Soc. [Ser. B]. 57, 289–300 10.2307/2346101

[B5] Bent S. J. Forney L. J. (2008). The tragedy of the uncommon: understanding limitations in the analysis of microbial diversity. ISME J. 2, 689–695 10.1038/ismej.2008.44 18463690

[B62] Berry D. Ben Mahfoudh K. Wagner M. Loy A. (2011). Barcoded primers used in multiplex amplicon pyrosequencing bias amplification. Appl. Environ. Microbiol. 77, 7846–7849 10.1128/AEM.05220-11 21890669 PMC3209180

[B6] Berry D. Stecher B. Schintlmeister A. Reichert J. Brugiroux S. Wild B. (2013). Host-compound foraging by intestinal microbiota revealed by single-cell stable isotope probing. Proc. Natl. Acad. Sci. U.S.A. 110, 4720–4725 10.1073/pnas.1219247110 23487774 PMC3607026

[B7] Bowers R. M. Lauber C. L. Wiedinmyer C. Hamady M. Hallar A. G. Fall R. (2009). Characterization of airborne microbial communities at a high-elevation site and their potential to act as atmospheric ice nuclei. Appl. Environ. Microbiol. 75, 5121–5130 10.1128/AEM.00447-09 19502432 PMC2725505

[B8] Briones A. Raskin L. (2003). Diversity and dynamics of microbial communities in engineered environments and their implications for process stability. Curr. Opin. Biotechnol. 14, 270–276 10.1016/S0958-1669(03)00065-X 12849779

[B9] Caporaso J. G. Lauber C. L. Walters W. A. Berg-Lyons D. Lozupone C. A. Turnbaugh P. J. (2011). Global patterns of 16S rRNA diversity at a depth of millions of sequences per sample. Proc. Natl. Acad. Sci. U.S.A. 108(Suppl. 1), 4516–4522 10.1073/pnas.1000080107 20534432 PMC3063599

[B10] Case T. J. (2000). An Illustrated Guide to Theoretical Ecology. New York, NY: Oxford University Press

[B61] Chandler D. P. Fredrickson J. K. Brockman F. J. (1997). Effect of PCR template concentration on the composition and distribution of total community 16S rDNA clone libraries. Mol. Ecol. 6, 475–482 10.1046/j.1365-294X.1997.00205.x 9161015

[B11] Cody M. L. Diamond J. M. (1975). Ecology and Evolution of Communities. Cambridge, MA: Harvard University Press

[B12] Csardi G. Nepusz T. (2006). The Igraph Software Package for Complex Network Research. Available online at: http://www.necsi.edu/events/iccs6/papers/c1602a3c126ba822d0bc4293371c.pdf

[B13] Curtis T. P. Sloan W. T. (2004). Prokaryotic diversity and its limits: microbial community structure in nature and implications for microbial ecology. Curr. Opin. Microbiol. 7, 221–226 10.1016/j.mib.2004.04.010 15196488

[B14] Eiler A. Heinrich F. Bertilsson S. (2012). Coherent dynamics and association networks among lake bacterioplankton taxa. ISME J. 6, 330–342 10.1038/ismej.2011.113 21881616 PMC3260505

[B15] Erdös P. Rényi A. (1959). On random graphs. Publ. Math-Debrecen 6, 290–297 20808879

[B16] Faith J. J. McNulty N. P. Rey F. E. Gordon J. I. (2011). Predicting a human gut microbiota's response to diet in gnotobiotic mice. Science 333, 101–104 10.1126/science.1206025 21596954 PMC3303606

[B63] Farrelly V. Rainey F. A. Stackebrandt E. (1995). Effect of genome size and rrn gene copy number on PCR amplification of 16S rRNA genes from a mixture of bacterial species. Appl. Environ. Microbiol. 61, 2798–2801 7618894 10.1128/aem.61.7.2798-2801.1995 PMC167554

[B17] Faust K. Raes J. (2012). Microbial interactions: from networks to models. Nat. Rev. Microbiol. 10, 538–550 10.1038/nrmicro2832 22796884

[B18] Fierer N. Lennon J. T. (2011). The generation and maintenance of diversity in microbial communities. Am. J. Bot. 98, 439–448 10.3732/ajb.1000498 21613137

[B19] Foster K. R. Bell T. (2012). Competition, not cooperation, dominates interactions among culturable microbial species. Curr. Biol. 22, 1845–1850 10.1016/j.cub.2012.08.005 22959348

[B20] Freilich S. Zarecki R. Eilam O. Segal E. S. Henry C. S. Kupiec M. (2011). Competitive and cooperative metabolic interactions in bacterial communities. Nat. Commun. 2, 589 10.1038/ncomms1597 22158444

[B21] Friedman J. Alm E. J. (2012). Inferring correlation networks from genomic survey data. PLoS Comp. Biol. 8:e1002687 10.1371/journal.pcbi.1002687 23028285 PMC3447976

[B22] Gotelli N. J. McCabe D. J. (2002). Species co-occurrence: a meta-analysis of J. M. Diamond's assembly rules model. Ecology 83, 2091 10.1890/0012-9658(2002)083[2091:SCOAMA]2.0.CO;2

[B23] Hibbing M. E. Fuqua C. Parsek M. R. Peterson S. B. (2010). Bacterial competition: surviving and thriving in the microbial jungle. Nat. Rev. Microbiol. 8, 15–25 10.1038/nrmicro2259 19946288 PMC2879262

[B24] Huber J. A. Mark Welch D. B. Morrison H. G. Huse S. M. Neal P. R. Butterfield D. A. (2007). Microbial population structures in the deep marine biosphere. Science 318, 97–100 10.1126/science.1146689 17916733

[B25] Klemm K. Eguíluz V. M. (2002). Highly clustered scale-free networks. Phys. Rev. E. Stat. Nonlin. Soft Matter Phys. 65:036123 10.1103/PhysRevE.65.036123 11909181

[B26] Klindworth A. Pruesse E. Schweer T. Peplies J. Quast C. Horn M. (2013). Evaluation of general 16S ribosomal RNA gene PCR primers for classical and next-generation sequencing-based diversity studies. Nucleic Acids Res. 41, e1 10.1093/nar/gks808 22933715 PMC3592464

[B27] Klitgord N. Segrè D. (2010). Environments that induce synthetic microbial ecosystems. PLoS Comput. Biol. 6:e1001002 10.1371/journal.pcbi.1001002 21124952 PMC2987903

[B28] Krumsiek J. Suhre K. Illig T. Adamski J. Theis F. J. (2011). Gaussian graphical modeling reconstructs pathway reactions from high-throughput metabolomics data. BMC Syst. Biol. 5:21 10.1186/1752-0509-5-21 21281499 PMC3224437

[B29] Lemos L. N. Fulthorpe R. R. Roesch L. F. W. (2012). Low sequencing efforts bias analyses of shared taxa in microbial communities. Folia Microbiol. 57, 409–413 10.1007/s12223-012-0155-0 22562492

[B30] Lozupone C. A. Hamady M. Kelley S. T. Knight R. (2007). Quantitative and qualitative beta diversity measures lead to different insights into factors that structure microbial communities. Appl. Environ. Microbiol. 73, 1576–1585 10.1128/AEM.01996-06 17220268 PMC1828774

[B31] Martiny A. C. Treseder K. Pusch G. (2013). Phylogenetic conservatism of functional traits in microorganisms. ISME J. 7, 830–838 10.1038/ismej.2012.160 23235290 PMC3603392

[B32] Montoya J. M. Pimm S. L. Solé R. V. (2006). Ecological networks and their fragility. Nature 442, 259–264 10.1038/nature04927 16855581

[B33] Mounier J. Monnet C. Vallaeys T. Arditi R. Sarthou A.-S. Hélias A. (2008). Microbial interactions within a cheese microbial community. Appl. Environ. Microbiol. 74, 172–181 10.1128/AEM.01338-07 17981942 PMC2223212

[B34] Pester M. Bittner N. Deevong P. Wagner M. Loy A. (2010). A ‘rare biosphere’ microorganism contributes to sulfate reduction in a peatland. ISME J. 4, 1591–1602 10.1038/ismej.2010.75 20535221 PMC4499578

[B60] Polz M. F. Cavanaugh C. M. (1998). Bias in template-to-product ratios in multitemplate PCR. Appl. Environ. Microbiol. 64, 3724–3730 9758791 10.1128/aem.64.10.3724-3730.1998 PMC106531

[B35] Power M. Tilman D. Estes J. Menge B. (1996). Challenges in the quest for keystones. BioScience 46, 609–620 10.2307/1312990

[B36] Prettejohn B. J. Berryman M. J. McDonnell M. D. (2011). Methods for generating complex networks with selected structural properties for simulations: a review and tutorial for neuroscientists. Front. Comput. Neurosci. 5:11 10.3389/fncom.2011.00011 21441986 PMC3059456

[B37] Rodriguez-Brito B. Li L. L. Wegley L. Furlan M. (2010). Viral and microbial community dynamics in four aquatic environments. ISME J. 4, 739–751 10.1038/ismej.2010.1 20147985

[B38] Ruan Q. Dutta D. Schwalbach M. S. Steele J. A. Fuhrman J. A. Sun F. (2006). Local similarity analysis reveals unique associations among marine bacterioplankton species and environmental factors. Bioinformatics 22, 2532–2538 10.1093/bioinformatics/btl417 16882654

[B39] Saavedra S. Stouffer D. B. Uzzi B. Bascompte J. (2011). Strong contributors to network persistence are the most vulnerable to extinction. Nature 478, 233–235 10.1038/nature10433 21918515

[B40] Schwab C. Berry D. Rauch I. Rennisch I. Ramesmayer J. Hainzl E. (2014). Longitudinal study of murine microbiota activity and interactions with the host during acute inflammation and recovery. ISME J. 8, 1101–1114 10.1038/ismej.2013.223 24401855 PMC3996699

[B41] Shade A. Peter H. Allison S. D. Baho D. L. Berga M. Bürgmann H. (2012). Fundamentals of microbial community resistance and resilience. Front. Microbiol. 3:417 10.3389/fmicb.2012.00417 23267351 PMC3525951

[B42] Shokralla S. Spall J. L. Gibson J. F. Hajibabaei M. (2012). Next-generation sequencing technologies for environmental DNA research. Mol. Ecol. 21, 1794–1805 10.1111/j.1365-294X.2012.05538.x 22486820

[B43] Simon C. Wiezer A. Strittmatter A. W. Daniel R. (2009). Phylogenetic diversity and metabolic potential revealed in a glacier ice metagenome. Appl. Environ. Microbiol. 75, 7519–7526 10.1128/AEM.00946-09 19801459 PMC2786415

[B44] Soetaert K. Petzoldt T. Woodrow Setzer R. (2010). Solving differential equations in R: Package deSolve. J. Stat. Softw. 33, 1–25 Available online at: http://www.jstatsoft.org/v33/i09/paper20808728

[B58] Stecher B. Berry D. Loy A. (2013). Colonization resistance and microbial ecophysiology: using gnotobiotic mouse models and single-cell technology to explore the intestinal jungle. FEMS Microbiol. Rev. 37, 793–829 10.1111/1574-6976.12024 23662775

[B45] Stein R. R. Bucci V. Toussaint N. C. Buffie C. G. Rätsch G. Pamer E. G. (2013). Ecological modeling from time-series inference: insight into dynamics and stability of intestinal microbiota. PLoS Comput. Biol. 9:e1003388 10.1371/journal.pcbi.1003388 24348232 PMC3861043

[B46] Stone L. Roberts A. (1992). Competitive exclusion, or species aggregation? Oecologia 91, 419–424 10.1007/BF00317632 28313551

[B65] Sun D. L. Jiang X. Wu Q. L. Zhou N. Y. (2013). Intragenomic heterogeneity of 16S rRNA genes causes overestimation of prokaryotic diversity. Appl. Environ. Microbiol. 79, 5962–5969 10.1128/AEM.01282-13 23872556 PMC3811346

[B47] Sunagawa S. Mende D. R. Zeller G. (2013). Metagenomic species profiling using universal phylogenetic marker genes. Nat. Methods 10, 1196–1199 10.1038/nmeth.2693 24141494

[B48] Tap J. Mondot S. Levenez F. Pelletier E. Caron C. Furet J.-P. (2009). Towards the human intestinal microbiota phylogenetic core. Environ. Microbiol. 11, 2574–2584 10.1111/j.1462-2920.2009.01982.x 19601958

[B49] Thébault E. Fontaine C. (2010). Stability of ecological communities and the architecture of mutualistic and trophic networks. Science 329, 853–856 10.1126/science.1188321 20705861

[B50] Tringe S. G. von Mering C. Kobayashi A. Salamov A. A. Chen K. Chang H. W. (2005). Comparative metagenomics of microbial communities. Science 308, 554–557 10.1126/science.1107851 15845853

[B51] Trosvik P. Rudi K. Straetkvern K. O. Jakobsen K. S. Naes T. Stenseth N. C. (2010). Web of ecological interactions in an experimental gut microbiota. Environ. Microbiol. 12, 2677–2687 10.1111/j.1462-2920.2010.02236.x 20482738

[B52] Tyson G. W. Chapman J. Hugenholtz P. Allen E. E. Ram R. J. Richardson P. M. (2004). Community structure and metabolism through reconstruction of microbial genomes from the environment. Nature 428, 37–43 10.1038/nature02340 14961025

[B53] Watts D. J. Strogatz S. H. (1998). Collective dynamics of “small-world” networks. Nature 393, 440–442 10.1038/30918 9623998

[B54] Wimpenny J. W. T. Colasanti R. (1997). A unifying hypothesis for the structure of microbial biofilms based on cellular automaton models. FEMS Microbiol. Rev. 22, 1–16 10.1111/j.1574-6941.1997.tb00351.x

[B55] Wirth T. Falush D. Lan R. Colles F. Mensa P. Wieler L. H. (2006). Sex and virulence in *Escherichia coli*: an evolutionary perspective. Mol. Microbiol. 60, 1136–1151 10.1111/j.1365-2958.2006.05172.x 16689791 PMC1557465

[B56] Ze X. Duncan S. H. Louis P. Flint H. J. (2012). *Ruminococcus bromii* is a keystone species for the degradation of resistant starch in the human colon. ISME J. 6, 1535–1543 10.1038/ismej.2012.4 22343308 PMC3400402

[B57] Zinger L. Amaral-Zettler L. A. Fuhrman J. A. Horner-Devine M. C. Huse S. M. Welch D. B. M. (2011). Global patterns of bacterial beta-diversity in seafloor and seawater ecosystems. PLoS ONE 6:e24570 10.1371/journal.pone.0024570 21931760 PMC3169623

